# Analysis of the Influence Mechanism of CO_2_ Emissions and Verification of the Environmental Kuznets Curve in China

**DOI:** 10.3390/ijerph16060944

**Published:** 2019-03-15

**Authors:** Debin Fang, Peng Hao, Zhengxin Wang, Jian Hao

**Affiliations:** 1 School of Economics and Management, Wuhan University, Wuhan 430072, China; dbfang@whu.edu.cn; 2 School of Economics, Zhejiang University of Finance & Economics, Hangzhou 310018, China; zxwang@zufe.edu.cn; 3 School of Business, Fuyang Normal University, Fuyang 236037, China; stevenhoou@163.com

**Keywords:** CO_2_ emissions, panel gray incidence degree, EKC, development stage, growth rate

## Abstract

Changes in economic development stage and growth type will lead to variations in the CO_2_ emissions. Traditional empirical analysis of the variations often only considers the impact of influencing factors on CO_2_ emissions from a single dimension. Under the background of China’s economy transferring from high-speed growth to high-quality development, this paper comprehensively considers the characteristics of the relevant influencing factors under different development stages and growth rates, and further calculates the panel gray incidence degree between CO_2_ emissions and these influencing factors in eastern, central, and western China. Based on the different development conditions, corresponding benchmarks of the indicators for the three regions (eastern, western, and central China) are accordingly set, highlighting the unity as well as the uniqueness between different regions. Furthermore, this paper verifies the environmental Kuznets curve (EKC) in the three regions. The result shows that all the factors of per capita Gross Domestic Product (GDP), Energy Intensity, Urbanization Level, and Trade Openness have a high correlation with CO_2_ emissions in the three regions, in which CO_2_ emissions are all between the two inflection points of the inverted N-shaped model.

## 1. Introduction

In the last forty years of China’s reform and opening up, energy consumption has also continued to increase as the economy grows rapidly. Due to massive consumption of fossil fuels, CO_2_ emission volume in China has continually increased: from 20.75 billion tons in 1990 to 91.40 billion tons in 2016, according to the data released by the International Energy Agency (IEA). In 2007, China’s total CO_2_ emissions exceeded that of the United States, ranking the first in CO_2_ emissions in the world. Since the excessive emission of CO_2_ has become an issue all over the world, the Chinese government has also formulated corresponding emission reduction plans. At the Copenhagen climate summit in 2009, the Chinese government announced that by 2020, its CO_2_ emissions related to per unit of GDP would be 40–45% lower than that in 2005, and issued by the State Council in 2016, points that: by 2020, carbon dioxide emissions of each unit of GDP will drop 18%, compared to 2015, and the total amount will be under control [1].

CO_2_ is an unavoidable byproduct of energy conservation and economic development. Its emission is not only closely linked to economic development, but is also influenced by many other economic factors. At the same time, the relevant economic indicators influence CO_2_ emissions from two aspects: the total volume and the growth rate. Take GDP for example; in general, the higher the GDP is, the more final consumption there is, or the more service is provided, both of which will directly lead to greater CO_2_ emissions. However, different GDP growth rates usually bring about different increment of CO_2_ emissions, and similarly, when the GDP growth rate is the same, different GDP bases also generate different CO_2_ emissions. At present, since China is in a transitional era, transferring from the previous high-speed growth type to high-quality mode, major changes in many aspects of its economic composition will take place and these changes will inevitably have an impact on China’s CO_2_ emissions. This requires us to consider the total amount and growth rate of the influencing indicators when analyzing carbon emissions. The past empirical analyses of the influences on CO_2_ emissions, such as decomposing analysis, causality test, cointegration analysis, and panel regression, only focused on either the stock or the growth figures of related factors and rarely integrated them. However, in the context of China’s economic aggregate ranking being second in the world and shifting from high-speed economic growth to high-quality development, comprehensive consideration of the characteristics of the relevant influencing factors under different development stages and growth rates would be helpful to analyze the change process of CO_2_ emissions more accurately.

Therefore, this paper selects per capita GDP, Energy Intensity, Urbanization Level, and Trade Openness as the main factors that affect CO_2_ emissions, attempting to study the influence of these factors on CO_2_ emissions by means of panel gray incidence analysis. Through analyzing the panel data from different dimensions, differences in development level and growth rate are applied to set up connections between the influencing factors and CO_2_ emissions, and then the correlations between them are calculated, which lays a more solid theoretical foundation for accurately describing the relationship between CO_2_ emissions and economic development. On this basis, taking Fixed Capital Investment, Energy Intensity, Urbanization Level, and Trade Openness as control variables and adding the third power of per capita GDP into the environment Kuznets curve (EKC) equation, we verify and analyze the panel EKC between economic growth and CO_2_ emissions in China during 1995 to 2016.

## 2. Literature Review

This part summarizes the existing research results from two aspects: We summarize the existing research results on the influencing factors of CO_2_ emissions, analyze and summarize the influencing factors of CO_2_ emissions obtained by other scholars through empirical analysis, and provide a theoretical basis for the selection of relevant factors in the empirical part of this paper. Secondly, we sort out the relevant research on the relationship between economic growth and CO_2_ emissions. These studies mainly reveal the relationship between the two through theoretical and empirical analysis, and analyze the relationship between the two from such aspects as economic growth theory, environmental economics, and energy economics.

### 2.1. Analysis of Influencing Factors of CO_2_ Emissions

When it comes to the study of influencing factors of CO_2_ emissions, the mainstream research method is decomposition analysis, which is mainly composed of index decomposition analysis (IDA) and structural decomposition analysis (SDA). In addition, scholars have been trying to find other ways to analyze CO_2_ emissions. Xu et al. [2] examine the impact factors of energy-related CO_2_ emissions in China from 1992 to 2011 using the Log Mean Divisia Index (LMDI), and empirical results indicate that economic output, population, and energy structure effects play an important role in the growth of carbon emissions. However, energy intensity has an obvious inhibitory effect on CO_2_ emissions. Compared to the good performance of other sectors in reducing carbon emissions, the CO_2_ emissions produced by the industrial sector have always shown a continuous upward trend. Fernández González et al. [3] decompose CO_2_ emissions in the European Union power sector into population, per capita output, fuel mix, carbonization, and energy intensity effects using the LMDI method. The results show that fuel mix has a strong impact on the level of total CO_2_ emissions, both overall and at country level, while the degree of impact is different in different countries. Using an extended STIRPAT model, Wang et al. [4] investigated some impact factors of CO_2_ emissions in Guangdong Province, China, and found that factors such as population, urbanization level, GDP per capita, industrialization level, and service level can cause an increase in CO_2_ emissions. However, technology level, energy consumption structure, and foreign trade degree can lead to a decrease in CO_2_ emissions. The estimated elastic coefficients suggest that population is the most important impact factor of CO_2_ emissions. Zhang and Lin [5] applied the STIRPAT model to analyze the relationship between urbanization level and carbon dioxide emissions and energy consumption in China from 1995 to 2010, and found that the effect of urbanization on energy consumption varied from region to region, and the effect is declining from the western region to the central and eastern regions. Wang et al. [6] studied the effect of socioeconomic factors, spatial planning factors, and transport networks on CO_2_ emissions in megacities in China based on the STIRPAT model by taking Beijing, Tianjin, Shanghai, and Guangzhou as samples and found that economic growth, urbanization, and industrialization would all lead to an increase in CO_2_ emissions, while the improvement of the level of equipment and technology could reduce CO_2_ emissions. Li et al. [7] divided China into five emission regions according to the annual per capita CO_2_ emissions, and used the STIRPAT model to analyze the influencing factors of CO_2_ emissions, and then found that per capita income, industrial structure, population, urbanization level, and technological level are all the major factors that affect the emission of carbon dioxide. However, in different regions, the effectiveness of each variable is different. Alam et al. [8] applied the Autoregressive Distributed Lag bounds test (ARDL) to analyze four emerging countries with the highest CO_2_ emissions (Brazil, China, India, and Indonesia). Empirical analysis shows that income and energy consumption are the most important influencing factors influencing CO_2_ emissions in the four countries. However, the population has a significant impact on CO_2_ emissions in India and Brazil, but the impact on China and Indonesia is insignificant. Rafindadi [9] applied causal analysis to study the relationship between CO_2_ emissions and financial development and energy consumption in Nigeria. Based on the ARDL model and the VECM model, it was concluded that the financial development would stimulate energy demand but could reduce CO_2_ emissions, while economic growth would reduce energy demand but increase CO_2_ emissions. Zhu et al. [10] applied the panel quantile regression model to analyze the impact of foreign direct investment (FAI), economic growth, and energy consumption on carbon emissions in the ASEAN-5 countries. The empirical analysis showed that the higher quantile of FAI has a significant negative impact on carbon emissions, while the higher quantile of energy consumption has a stronger effect on CO_2_ emissions growth. The increase in trade openness can ease the increase of carbon emissions in both low and high CO_2_ emission scenarios. Pao and Tsai [11] applied the panel cointegration technique to analyze the relationship between CO_2_ emissions and GDP, energy consumption, and FAI in BRIC countries, and found that the four variables have a long-term equilibrium relationship and support the EKC hypothesis in the four countries. Meanwhile, CO_2_ emissions are energy consumption elastic and FAI inelastic, and suggest that developing countries should step up their technology transfer when they cooperate with foreign countries and avoid becoming a pollution haven. Abid [12] used the GMM dynamic panel model to analyze the influencing factors of carbon dioxide emissions in the 25 sub-Saharan countries, showing that these countries do not support the EKC hypothesis and carbon emissions in these countries show a monotonous growth trend with economic growth. At the same time, political factors also have a significant impact on CO_2_ emissions: political stability, higher government efficiency, promotion of democracy, and corruption control can help reduce CO_2_ emissions. Through the SDA-EIOT model, Liang and Zhang [13] analyzed the factors that affect carbon emissions in Jiangsu Province, the manufacturing center of Southern China, pointing out that Jiangsu Province should not only focus on the reduction of energy consumption intensity and the optimization of energy consumption structure, but also pay more attention to the decline of hidden carbon in international export trade. By weighing the geographic information and energy consumption changes of the provinces, Dong et al. [14] analyzed the correlation between industrial pollution and CO_2_ emissions in eastern, western, and central China, pointing out that industrial carbon emissions play a significant role in promoting the increase of carbon density. Due to spatio-temporal heterogeneity, this kind of promoting effect differs between provinces, while the estimated parameter results of neighboring provinces are not substantially different. Based on the estimation of the carbon emissions efficiency of China’s 30 provinces, Wang et al. [15] found that whether directly or indirectly, resource abundance has an inhibitory effect on the improvement of carbon emission efficiency. Li et al. [16] analyzed the impact of natural resource abundance on China’s industrial structure and carbon emissions, and the results showed that the rationalization of manufacturing structure was not only conducive to reducing the intensity of carbon emissions, but also helpful to weaken the limits of natural endowments. Li et al.’s [17] analysis of carbon emissions of more than 8000 households in 25 provinces in China pointed out that the subjective factors of households had a significant impact on carbon emissions, among which feeling secure, compliance with rules, and happiness had remarkable inhibitory effects, while the attention to social issues did not promote family members to consciously reduce carbon emissions. Information spillover has an effect on carbon emissions [18,19].

It was found that although scholars generally believed that CO_2_ emissions are influenced by many factors with different outcomes, previous studies, no matter the regression analysis or indicators’ decomposition, often only considered indicators from a single dimension when it comes selecting indicators or factor decomposition. However, as China’s economic aggregates have been increasing and the country is paying more attention to high-quality development, simply taking the total amount of relevant indicators or growth rate of relevant indicators into consideration cannot accurately describe the current economic situation. The advantages of gray incidence analysis are that it could better integrate the development level and growth rate and analyze the correlation between relevant factors and CO_2_ emissions from the two dimensions. Therefore, gray incidence analysis can analyze the mechanisms of CO_2_ emissions more comprehensively.

### 2.2. Relation between Economic Growth and CO_2_ Emissions

Since Grossman and Kruegei [20] introduced the Kuznets curve into the study of economic growth quality and got the inverted U-shape relation between economic growth and environmental pollution, the environmental Kuznets curve, also known as EKC, has gradually become one of the most important tools for scholars to explain the relation between economic growth and CO_2_ emissions. Then, Shafik [21] and Selden and Song [22] verified the inverted U-shape relation that exists between per capita GDP and environment quality. In recent years, most scholars apply FMOLS, DOLS, ARDL, and other methods to verify the inverted U-shaped relation between economic growth and environmental pollution [8,23,24,25,26] based on data from different countries. However, some scholars have pointed out that the EKC does not exist between economic growth and environmental pollution [27,28,29]. With the continuous expansion of research, scholars have used different sample data to verify the relationship between economic growth and environmental pollution, and find that there are diverse results. Zarzoso and Morancho [30] found that there was an N-shaped relationship between per capita CO_2_ emissions and per capita GDP by means of analyzing data from 22 OECD countries. Meanwhile, a decoupling situation appeared between CO_2_ emission and economic growth in some sample countries. Kang et al. [31] used the spatial panel data model to examine the EKC hypothesis of China’s CO_2_ and the results showed that the relationship between economic growth and CO_2_ emission is an inverted N shape. Olugbenga and Onafowora [32] analyzed multinational data and found that the inverted U-shaped EKC hypothesis was true in Japan and South Korea while the long-term relationship between economic growth and CO_2_ emissions was N-shaped in Brazil, China, Egypt, Mexico, Nigeria, and South Asia. Meng et al. [33] used the LMDI method to decompose carbon emissions into seven effects from the view of economy, energy, and science and technology input, empirically analyzing the carbon emissions of various provinces and cities in China. The results indicated that economic growth and investment growth were the most important factors for the rise of carbon emissions, while the increase in R&D investment was conducive to curbing it.

Summarizing the existing studies, we can see that scholars generally believe there is a nonlinear relationship between economic growth and CO_2_ emissions. Because of the differences in regions, time spans, indicators, and models, however, it is difficult to draw an agreed conclusion on what kind of nonlinear relationship exists between carbon emissions and economic growth. As for the study of the relationship between China’s economic growth and CO_2_ emissions, the focus of controversy is mainly laid on whether there is a relationship between them (that is, which line type it is, inverted U-type, N-type, or inverted N-type). The existence of such problems has led to disagreements among scholars on whether China will reach the inflection point of CO_2_ emissions. Therefore, this paper adds the third power of GDP while modeling to validate the EKC hypothesis in China and, furthermore, to test whether there is an N or -inverted N-type relationship between economic growth and CO_2_ emissions.

## 3. Modeling and Empirical Analysis of Panel Gray Relation

The advantages of the gray incidence method are that it has a lower requirement of data characteristics but has good accuracy when it comes to time- and sample-limited research. Moreover, based on unifying the observation features of different dimensions, it can more comprehensively and accurately describe the correlation between samples [34,35,36]. With the widespread use of panel data in recent years, gray incidence analysis also has extended from the initial analysis of plane curves to three-dimensional spatial relationship analysis [37,38,39].

### 3.1. Modeling of Panel Gray Incidence Analysis

$x_{i}(m,t)$ is set as the value of research sample *i* at time *t* considering indicator *m* in the panel data, where *i* = 1, 2, …, *N*; *m* = 1, 2, …, *M*; *t* = 1, 2, …, *T*. Before the calculation, this paper chooses the initial operator processing method to solve the dimension problems of multiple variables [34]. In the process of panel gray incidence analysis, we first need to calculate the value of dissimilarity degree. Dissimilarity degree reflects the level of the dissimilarity of the two variables. When the dissimilarity degree of the two variables is relatively small, it indicates their changing trends towards the same direction, that is, their similarity is high. To measure the dissimilarity degree of the cross-sectional dimension between two research samples, we apply the dissimilarity degree of development level index.

Let *i* and *j* be research objects,

(1) $$dis_{ij}^{1}\left(m,t\right)=\frac{\Delta_{ij}\left(m,t\right)}{x_{j}\left(m,t\right)}$$

where $dis_{ij}^{1}\left(m,t\right)$ is called the dissimilarity degree of development level of i and j, and $\Delta_{ij}\left(m,t\right)=x_{i}\left(m,t\right)-x_{j}\left(m,t\right)$, $\Delta_{j}\left(m,t\right)=x_{j}\left(m,t\right)-x_{j}\left(m,t-1\right)$.

$\Delta_{i}\left(m,t\right)$ and $\Delta_{j}\left(m,t\right)$ reflect the increment of two sequential development periods of objects *i* and *j*. If several indicators of the two objects are increasing in the same direction at the same or a similar growth rate, then the dissimilarity degree of dynamic growth is small; on the contrary, if there is a large growth difference among several indicators, even in opposite directions, then the dissimilarity degree is big. Then

(2) $$\alpha_{ij}^{1}\left(m,t\right)=\frac{1}{1+\left|dis_{ij}^{1}\left(m,t\right)\right|}$$

(3) $$\alpha_{ij}^{2}\left(m,t\right)=\frac{1}{1+\left|dis_{ij}^{2}\left(m,t\right)\right|}$$

where $\alpha_{ij}^{1}\left(m,t\right)$ represents the associated sequence of the development level, $\alpha_{ij}^{2}\left(m,t\right)$ represents the associated sequence of growth. Therefore, the gray incidence degree of development level between matrix $x_{i}$ and $x_{j}$ can be calculated.

The gray incidence degree of the development level:

(4) $$\beta_{ij}^{1}=\frac{1}{T}\sum_{t=1}^{T}\alpha_{ij}^{1}\left(m,t\right)$$

The gray incidence degree of the growth level:

(5) $$\beta_{ij}^{2}=\frac{1}{T-1}\sum_{t=2}^{T}\alpha_{ij}^{2}\left(m,t\right)$$

Further, the integrated panel gray incidence degree between matrix $x_{i}$ and $x_{j}$ is

(6) $$\beta_{ij}=\left(\beta_{ij}^{1}+\beta_{ij}^{2}\right)/2$$

Then, the order of these variables can be drawn by comparing the integrated panel gray incidence degree.

### 3.2. Empirical Analysis of Panel Gray Relation

#### 3.2.1. Data Sources

This paper applies the material balance method recommended by the IPCC (Intergovernmental Panel on Climate Change) to calculate CO_2_ emissions from 1995 to 2016 in China. It is based on the law of conservation of energy in the process of energy consumption. The volume of CO_2_ emissions is calculated by counting, discounting, and totaling primary and secondary energy in all sectors in an economic system, the basic formula of which is as follows:

(7) $${\mathrm{CO}}_{2_{i}}=\sum_{j}^{k}c_{ij}\cdot m_{{\mathrm{co}}_{2}}/m_{c}=\sum_{j}^{k}E_{ij}F_{ij}\cdot 44/12$$

where CO_2_ (Note: Due to the limit of length, we could not present a specific value here. Interested readers can ask for it from us) represents CO_2_ emissions of inland province *i* in China (except Tibet), i = 1, 2, …, 30. *j* (*j* = 1, 2, …, 8) represents 8 kinds of fossil fuels: coal, coke, crude oil, gasoline, kerosene, diesel, fuel oil, and natural gas. $m_{c}$ and $m_{{\mathrm{co}}_{2}}$ respectively represent the relative atomic mass of carbon atoms and the relative molecular mass of CO_2_, which are 12 and 44. $E_{ij}$ is the consumption of the energy *j* in region *i*, and $F_{ij}$ is the emissions coefficient of the energy *j* in province *i*. the values of coefficient come from the guidelines for the compilation of provincial greenhouse gas inventories [40].

We selected the five variables of per capita GDP, Energy Intensity, Urbanization Level, and Trade Openness and analyzed the gray incidence degree between them and China’s per capita CO_2_ emissions. All variables are mainly from the Yearbook of China Statistical Yearbook, the National Bureau of Statistics, the China Energy Statistical Yearbook, and the Statistical Yearbook of each province and city of China.

(a). Per Capita GDP (*RGDP*): According to the EKC hypothesis, economists generally believe that changes in income levels will have an important impact on the environment. Therefore, in this paper, we choose the per capita GDP to represent the income level. In order to eliminate the impact of price factor, this paper regards the price in 1995 as the price of the base year and separately makes price adjustments and takes the logarithm of the relevant data. Unit: yuan/person.

(b). Energy Intensity (*EI*): Similarly, in an extended production function, energy is another important factor affecting economic growth. Coal, because of limited natural resources in China, has dominated throughout China’s energy structure, which has released a great deal of CO_2_. Recently, as production technology has significantly advanced, enterprises can create more value while conserving less energy. Therefore, this paper chooses the fluctuation of energy intensity to represent the technology level. Unit: ton/10^4^ yuan.

(c). Urbanization Level (*UL*): The rise in the level of urbanization has expanded the size of the cities, increased the population, and created more industry, services, and CO_2_ emissions. Due to the imbalance of economic development in different regions in China, the corresponding level of economic development is quite different. In this paper, urbanization level is represented by the proportion of urban population in total population. Unit: %.

(d). Trade openness (*TO*): China produces a significant number of export products every year, but leaves the energy consumption and environmental pollution at home. This paper takes the proportion of total import and export volume in GDP to represent trade openness. Unit: %.

#### 3.2.2. Empirical Results of Panel Gray Incidence Analysis

According to China’s administrative region, this paper divides China into three parts: eastern China, central China, and western China. Then, based on the panel gray incidence model in Section 3.1, we calculate the gray incidence degree between per capita CO_2_ emissions and the four factors of per capita GDP, Energy Intensity, Urbanization Level, and Trade Openness in China from 1995 to 2016. Then the gray incidence degree of each factor is ranked in descending order and shown in Table 1.

According to the model established by Section 3.1, it can be seen that the closer the gray incidence degree gets to 1, the higher the correlation between the variables and CO_2_ emissions. It can be seen from Table 1 that the gray incidence value of each influencing factor in each region is high, which indicates that the difference between the influencing factors and the per capita CO_2_ emissions is low, that is, the similarity is high. According to Section 3.1, since the grey incidence degree is a weighting of the level of development and the growth rate, CO_2_ emissions in these regions are not only related to the stock of these indicators, but also to the rate of change of these indicators. We will provide further explanation in Section 4.

**Figure 1:**
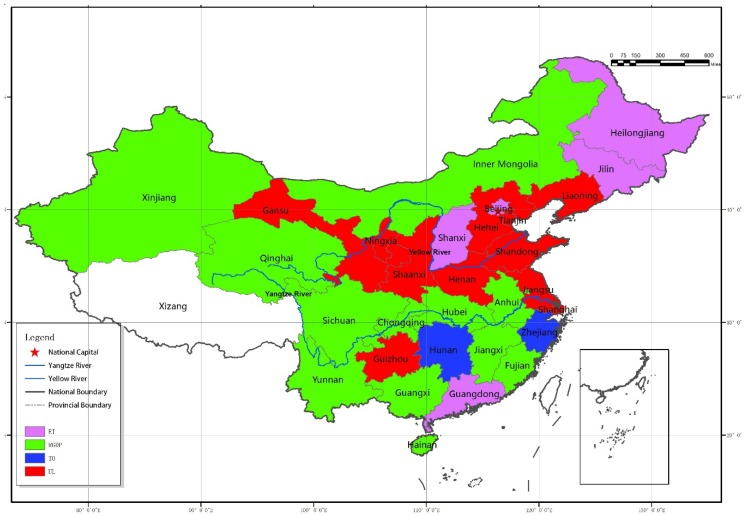
Regional distribution of primary influencing factors of CO_2_ emissions (Note: Due to the lack of data, the Tibet region is depicted as blank.).

At the national level, there are 12 regions (Fujian, Hainan, Anhui, Jiangxi, Hubei, Inner Mongolia, Guangxi, Chongqing, Sichuan, Yunnan, Qinghai, and Xinjiang) showing that per capita GDP is the biggest gray incidence indicator, and there are 11 regions (Tianjin, Hebei, Liaoning, Shanghai, Jiangsu, Shandong, Henan, Guizhou, Shaanxi, Gansu, and Ningxia) showing that Urbanization Level is the biggest gray incidence indicator among influential factors. In addition, Energy Intensity in Beijing, Guangdong, Shanxi, Jilin, and Heilongjiang shows the largest gray incidence degree, while Trade Openness in Zhejiang and Hunan demonstrates the largest gray incidence degree. At the regional level, gray incidence degree between these factors shows strong regional characteristics. From the average value of gray incidence degree of all factors in the three regions, it can be seen that eastern region ranks the highest with 0.8177; followed by the central region (0.8091); then the western (0.7485). This means that each factor has a higher similarity with CO_2_ emissions in eastern region, then the central, and finally the western. At the same time, the grey incidence degree of these indicators is different among different regions. Take, for example, the factor with the highest incidence degree (Figure 1). Among the 11 provinces and cities in the eastern region, more than half show that *UL* is the factor with the highest incidence degree. Among the eight provinces and cities in the central region, six show *RGDP* and *EI* to have the highest incidence degree. Among the 11 provinces and cities in the western region, *RGDP* ranks the first in seven and *UL* is the highest in the rest. In addition, note that due to the lower trade openness in the western region than that in the eastern and central regions, *TO* has the lowest gray incidence level in almost all the western provinces and cities (except Guizhou).

## 4. Verification of EKC for CO_2_ Emissions in China

The analysis of Section 3 shows that the four factors have a high gray incidence with CO_2_ emissions, which indicates that they are all important factors affecting CO_2_ emissions. However, due to the economic discrepancy among the different regions, influencing factors also have different performances. Therefore, this part establishes the EKC equation for Eastern China, Central China, and Western China, analyzing the relationship between economic development and China’s CO_2_ emissions, then testing the impact of the related factors on CO_2_ emissions.

### 4.1. Model Establishment

In the classical EKC empirical study, scholars tend to apply pollutants as explained variables, and GDP as the single explanatory variable. However, when the level of national income changes, usually some other economic indicators also change, such as technical level, the level of urbanization, trade openness, and so on. Therefore, in order to more accurately measure the effect of other variables on per capita CO_2_ emissions during the analysis of the dynamic relation between CO_2_ emissions and per capita GDP, this paper adds control variables including *EI*, *UL*, and *TO* to analyze the relation between the two with the provincial panel data from 1995 to 2016. Meanwhile, in classic studies, whether the explanatory variables have a U-type or inverted U-type relation with the explained variable is mainly confirmed by observing the coefficient of the quadratic term. However, some scholars hold that there should be more complicated three-curve relations than simple U-shaped and inverted U-shaped types. This paper attempts to add the third power of per capita GDP to the regression equation. The model is as follows:

(8) $$\begin{array}{cc} {\mathrm{LnRCO}}_{2it} & =\alpha +\beta_{1}\mathrm{Ln}RGDP_{it}^{3}+\beta_{2}\mathrm{Ln}RGDP_{it}^{2}+\beta_{3}\mathrm{Ln}RGDP_{it}\\ & +\beta_{4}\mathrm{Ln}EI_{it}+\beta_{5}\mathrm{Ln}UL_{it}+\beta_{6}\mathrm{Ln}TO_{it}+\varepsilon_{it} \end{array}$$

where, *i* represents region *i*, *t* represents the year. RCO_2_ is per capita CO_2_ emissions. In order to eliminate the effect of heteroscedasticity to the results, all variables are taken logarithmically.

### 4.2. Assumption Analysis of Verification Results

Based on Equation (8), the results of the model may appear as different situations, as listed in Table 2. It can be seen that the difference of the cubic terms and the quadratic terms of the per capita CO_2_ emissions in the model determine the different nonlinear relation between per capita CO_2_ emissions and per capita GDP. In the case when the cubic term of per capita CO_2_ emissions is not zero, an N-type or inverted N-type relation between the two may appear. When the cubic term is zero but the quadratic term is not zero, the relation may express a U-shaped or inverted U-type.

### 4.3. Empirical Result of EKC Model

The fixed effect model (FE) and the random effect model (RE) are the common estimation methods for panel data regression [41]. In this section, the Hausman Test is used to test which one is better but due to the limitation of length, but this part is omitted. Finally, the FE method is chosen to analyze CO_2_ emissions in three regions. The regression results of the three regions are as follows (Table 3):

As can be seen from Table 3, the coefficient of $RGDP^{3}$, $RGDP^{2}$, RGDP, and other control variables pass the significance test. R^2^ values of Eastern China, Central China, and Western China are 0.854, 0.962, and 0.877, respectively, which indicates that the model can explain CO_2_ emissions well in the three regions. This result shows that taking per capita GDP, Energy Intensity, Urbanization Level, and Trade Openness as explanatory variables can explain the changes of per capita CO_2_ emissions in the three regions. According to mathematical knowledge, the inflection points of EKC and line-type in three regions are as follows:

As shown in Table 4, EKC demonstrates the inverted-N type in all the three regions, implying that as per capita GDP increases, per capita CO_2_ emissions decrease first, then increase and then decrease again. Meanwhile, the results from the derivative of the EKC equation show that two inflection points exist in every region and their value are both positive numbers, indicating that they have realistic economic significance: as per capita GDP increases, per capita CO_2_ emissions decrease before the first inflection point, increase when in the middle between the two points and decrease again after passing the second inflection point.

**Figure 2:**
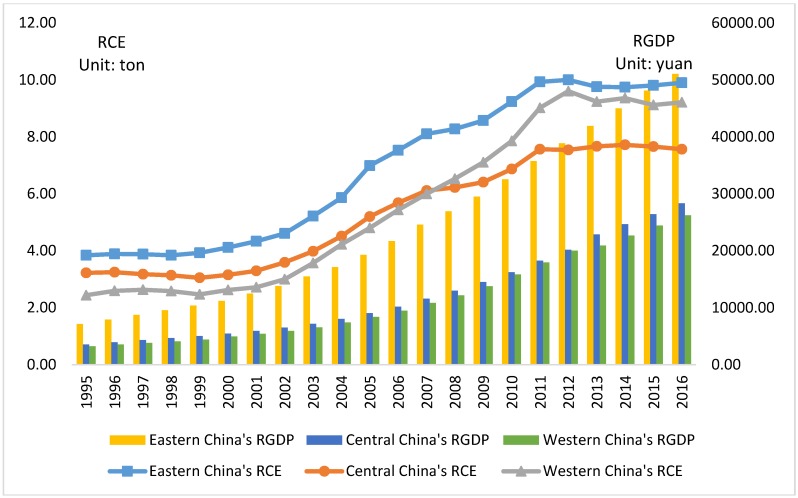
1995–2016 per capita GDP (*RGDP*) and per capita CO_2_ emissions (*RCE*) of eastern, central, and western China.

Combined with the economic reality (Figure 2), it can be checked that in 1995, *RGDP*_eastern,1995_ = 7162.11, *RGDP*_central,1995_ = 3578.78, and *RGDP*_western,1995_ = 3271.27, all of which exceeded the first turning point. With continuous economic advancement, in 2016, the *RGDP* in three regions reached *RGDP*_eastern,2016_ = 51,065.76, *RGDP*_central,2016_ = 28,363.56, and *RGDP*_western,2016_ = 26,254.39, all of which were less than the second inflection point, signifying that during the sample period, per capita CO_2_ emissions in all three regions were increasing as per capita GDP increased. It also can be found that the per capita GDP in the eastern region is approaching the second point, implying that with further development, per capita CO_2_ emissions in the eastern region are likely to show a decrease.

**Figure 3:**
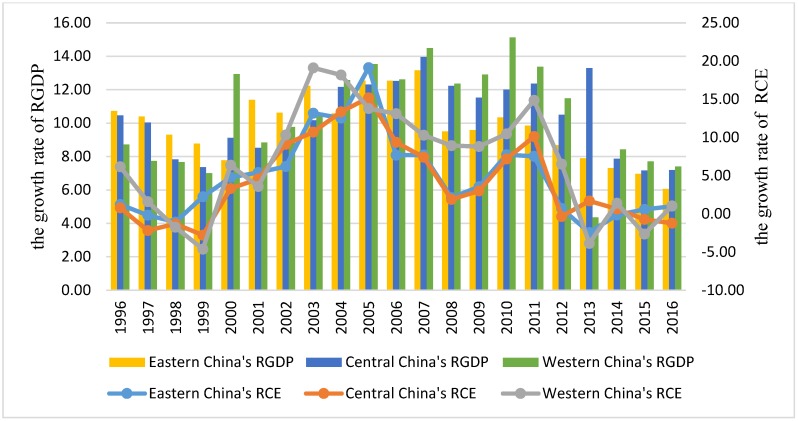
The growth rates of *RGDP* and RCE in the eastern, central, and western China during 1995–2016.

Combined with the analysis in Section 3, the findings can be explained from the perspectives of development level and growth rate of per capita GDP. After comparing the actual value (Figure 2) and change rate (Figure 3) of per capita CO_2_ emissions and per capita GDP of the three regions, it can be seen that the change trends of per capita CO_2_ emissions as well as per capita GDP in eastern, central, and western China basically kept the same pace from 1995 to 2016. The reason was that in 1992, the Chinese government set the goal of reform of “establishing a socialist market economy system”. Driven by the reform and opening up, China’s CO_2_ emissions have risen again since 1993, when the first inflection point of China’s CO_2_ emissions began to appear. After that, China’s per capita CO_2_ emissions have gradually increased with the growth of *RGDP*, demonstrating an overall rising tendency. In particular, after China’s accession to the WTO in 2000, the Chinese economy entered a period of rapid growth and its total GDP increased from 9.38 trillion in 2000 to 20.45 trillion in 2007, which has led to a significant increase in energy consumption and CO_2_ emissions. Due to the impact of the US financial crisis in 2008, China’s economic growth rate declined, and the growth of CO_2_ emissions also slowed down. At present, China’s economic growth rate eased. During the “Eleventh Five-Year Plan” (2006–2010), China’s average annual economic growth rate was 11.3% and during the “Twelfth Five-Year Plan” (2011–2015), it was 7.8%. The annual economic growth rate during the “Thirteenth Five-Year Plan” period (2016–2020) is expected to drop to 7%. The slowdown in economic growth has also led to a decline in the growth rate of CO_2_ emissions. At the same time, the Chinese government began to enhance the protection of environment. In 2014, the amendment of the “Environmental Protection Law” was issued, and laws on atmospheric protection and water pollution control were released in succession, which also promoted the reduction of CO_2_ emissions. Although China’s per capita CO_2_ emissions peaked during the “Twelfth Five-Year Plan”, the overall downward trend remains slow. In China’s current CO_2_ emissions mechanism, reducing factors and increasing factors coexist, and emission levels are subject to uncertainties in economic growth and the strictness and sustainability of environmental policies, which may cause the inflection point of environmental pollution be characterized by fluctuating about a high level. Therefore, although China’s per capita CO_2_ emissions are getting close to the inflection point, its suspended time at the inflection point peak may be relatively long, delaying comprehensively improved environmental quality.

At the same time, from the regional point of view, the inflection point of environmental quality is not consistent at all time points in China. In general, the emissions of traditional pollutants in the eastern region has reached and crossed the peak already, but the improvement of environmental quality is still relatively slow. The central region is currently at the peak of the environmental Kuznets curve, and its environmental quality is at a minimum. With the further advancement of urbanization, its suspended time at the peak may be relatively long. The western region as a whole is still in the climbing phase of the environmental Kuznets curve, and the pollutant emissions during industrialization remain the main source of future environmental pressures.

**Figure 4:**
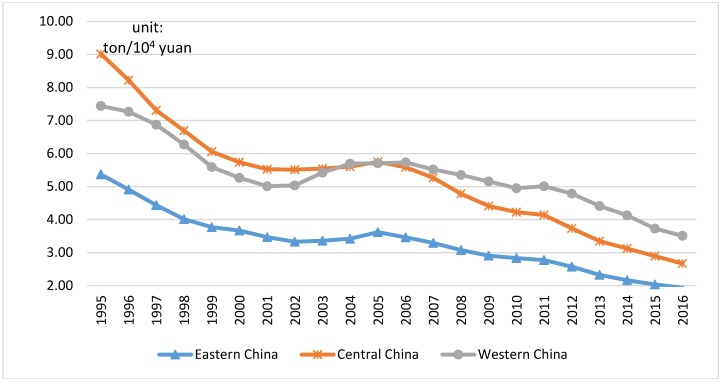
1995–2016 energy intensity of eastern, central, and western China.

At the same time, it can be also seen from Table 3 that *EI*, *UL*, and *TO* have different impacts in different regions. In all the three regions, the coefficient of *EI* is positive, which indicates that the increase in energy intensity will lead to an increase in CO_2_ emissions, that is, the decrease in energy intensity is beneficial to suppressing the increase of CO_2_ emission. As can be seen from Figure 4, from 1995 to 2016, the energy intensity of China’s eastern, central, and western regions was in an overall decline, down by 63.87%, 70.39%, and 52.82%, respectively. Combined with the regression coefficient, it can be seen that the reduction of energy intensity in the eastern region has the strongest inhibitory effect on its carbon emissions: one unit of energy intensity decreases, 0.65 tons CO_2_ emissions can be reduced. Similarly, each unit reduction of energy intensity in the central and western regions could bring 0.27-ton and 0.54-ton decreases in CO_2_ emissions, respectively. Meanwhile, the improvement of urbanization level increases per capita CO_2_ emissions in eastern and central China but reduces that of the western region. The level of urbanization has a stronger role in promoting the increase of per capita carbon dioxide emissions in the eastern region than in the central. For every 1% increase in urbanization level, the per capita carbon dioxide emissions will increase by 1.051 tons in the eastern while 0.546 tons in the central region. However, the increase in urbanization level is conducive to curbing the increase in carbon dioxide emissions in the western region. If the urbanization level in the western region increases by 1%, it will effectively reduce the per capita carbon dioxide emissions by 0.226 tons. The increase in trade openness reduces per capita carbon dioxide emissions in the eastern and western regions, but increases that of the central region. A one percent increase in trade openness will exert an impact on per capita CO_2_ emissions in the three regions of −0.202 tons, 0.10 tons, and −0.144 tons, respectively.

In summary, economic growth will affect environmental quality through energy intensity, urbanization level, and trade openness. The main mechanism is as follow: as the economic scale expands and per capita GDP increases, the more energy is consumed, the more serious the environmental pollution is. With the continuous development of the economy, the upgrading of the production process improves the energy utilization efficiency and reduces the energy consumption intensity, effectively curbing the increase of carbon dioxide emissions. Although the improvement of urbanization brings about new requirements for infrastructure construction, such as urban residential areas and transportation, and the consumption of a large amount of energy will cause an increase in carbon dioxide emissions, as China’s urbanization process is gradually completed, its role in promoting carbon dioxide emissions is bound to weaken. At the same time, a reasonable urban layout and an efficient energy consumption system will play a role in reducing per capita carbon dioxide emissions by means of the city scale’s effect on population and energy consumption.

## 5. Suggestions

This paper applies the panel gray incidence analysis method to study the gray incidence degree between CO_2_ emissions and the four influencing factors of per capita GDP, Energy Intensity, Urbanization Level, and Trade Openness. It is found that all these factors have a relatively high correlation with CO_2_ emissions with regional characteristics. At the same time, on the basis of the result, we apply panel data to verify the EKC hypothesis, discovering that per capita CO_2_ emissions show an “inverted N-type” relation with per capita GDP in eastern, central, and western China. Moreover, per capita GDP in all these three regions is between the two inflection points, implying that per capita CO_2_ emissions will increase as per capita GDP increases.

For the sake of national economic development and to meet the needs of the people’s quality of life, the reduction of CO_2_ emissions can’t be realized at the expense of economic growth. However, the growth of CO_2_ emissions would be mitigated by improving the quality of economic development and optimizing the layout of urbanization. The main policy implications of this article are: (1) Advanced low carbon technology is the key to reducing the intensity of carbon emissions. For China, due to the limited research and development capabilities, the innovation in high-level low-carbon technologies is insufficient. Therefore, in order to improve the innovation level, China should first establish a market environment for independent innovation of low-carbon technologies and encourage and support small and medium-sized enterprises to carry out low-carbon technology innovation. Secondly, China should actively cooperate with countries with relatively mature low-carbon technologies, such as Japan, the United Kingdom, and Germany, and learn from them in technological areas, such as greenhouse gas measurement, low-carbon manufacturing, renewable energy manufacturing, and green energy storage, so as to make better use of global low carbon innovation resources. Finally, the government should strengthen the construction of the intellectual property system to provide a good protection environment for low-carbon technology innovation. (2) In the context of the Chinese government continuing to promote urbanization, if we want to maintain sustainable urban development and reduce carbon dioxide emissions, the traditional urbanization development model that relies on land and tax subsidies should be abandoned and low-carbon development needs to be explored. Human capital investment could improve people’s scientific and cultural quality and enhance people’s environmental literacy, having a positive effect on carbon emission reduction in China’s urbanization process. Furthermore, we should vigorously promote the development concept of “low-carbon cities” and give enterprises or individuals financial and tax support and policy incentives in new energy utilization and clean production. In urban transportation systems, there is huge potential for energy conservation. Rail transit construction should be actively promoted, and low-carbon vehicles, such as electric vehicles, hybrid vehicles, and fuel cell vehicles, should be encouraged. At the same time, to establish building laws and regulations based on energy-saving, research related to the design and evaluation of energy-efficient buildings should be supported, and the use of energy-saving materials should be increased. (3) At present, China is still the world’s largest import and export trade country. Although, after 2007, China’s industry structure of export trade has gradually shifted from mainly exporting primary goods to manufactured goods [42], China still needs to pay attention to reducing the proportion of low value-added traditional manufacturing products with high emissions in global trade, and develop general trade and service trade that is dominated by low-energy products coming from renewable energy, renewable materials, and newly-emerging industries, and further transform the export structure to high-tech, value-added industries that are low-energy and eco-friendly by means of continuous adjustment of trade policies. At the same time, as market demand is changing and new services are constantly emerging, China should expand its depth of service industries.

## 6. Conclusions

This paper applies the panel gray incidence analysis method to study the gray incidence degree between CO_2_ emissions and the four influencing factors of per capita GDP, Energy Intensity, Urbanization Level, and Trade Openness. It is found that all these factors have a relatively high correlation with CO_2_ emissions with regional characteristics. At the same time, on the basis of the result, we apply panel data to verify the EKC hypothesis, discovering that per capita CO_2_ emissions show an “inverted N-type” relation with per capita GDP in eastern, central, and western China. Moreover, per capita GDP in all these three regions is between the two inflection points, implying that per capita CO_2_ emissions will increase as per capita GDP increases.

## Figures and Tables

**Table 1:** Panel gray incidence ranking of influencing factors of CO_2_ emissions.

Regions	First	Second	Third	Fourth
Beijing	*EI*	*UL*	*RGDP*	*TO*
	0.9142	0.7981	0.7843	0.6736
Tianjin	*UL*	*TO*	*EI*	*RGDP*
	0.9151	0.8776	0.8586	0.8290
Hebei	*UL*	*EI*	*RGDP*	*TO*
	0.8364	0.8207	0.7977	0.7298
Liaoning	*UL*	*EI*	*RGDP*	*TO*
	0.8624	0.8484	0.8022	0.7562
Shanghai	*UL*	*TO*	*EI*	*RGDP*
	0.9492	0.8512	0.8268	0.8191
Jiangsu	*UL*	*RGDP*	*TO*	*EI*
	0.8924	0.8790	0.8242	0.7983
Zhejiang	*TO*	*RGDP*	*UL*	*EI*
	0.8994	0.8857	0.8781	0.8316
Fujian	*RGDP*	*UL*	*TO*	*EI*
	0.8066	0.7732	0.7410	0.7170
Shandong	*UL*	*RGDP*	*TO*	*EI*
	0.8759	0.8692	0.8074	0.7737
Guangdong	*EI*	*RGDP*	*UL*	*TO*
	0.8307	0.8294	0.7457	0.6213
Hainan	*RGDP*	*UL*	*TO*	*EI*
	0.8169	0.7330	0.6823	0.6519
Shanxi	*EI*	*UL*	*RGDP*	*TO*
	0.8140	0.7889	0.7655	0.7413
Jilin	*EI*	*UL*	*RGDP*	*TO*
	0.8724	0.8671	0.8455	0.8043
Heilongjiang	*EI*	*RGDP*	*UL*	*TO*
	0.8823	0.8694	0.8683	0.8509
Anhui	*RGDP*	*UL*	*EI*	*TO*
	0.8729	0.8434	0.7702	0.7211
Jiangxi	*RGDP*	*UL*	*EI*	*TO*
	0.8162	0.7330	0.7203	0.6768
Henan	*UL*	*RGDP*	*TO*	*EI*
	0.8958	0.8754	0.8579	0.7854
Hubei	*RGDP*	*EI*	*TO*	*UL*
	0.8531	0.8318	0.8128	0.8123
Hunan	*TO*	*RGDP*	*UL*	*EI*
	0.7914	0.7764	0.7434	0.7309
Inner Mongolia	*RGDP*	*UL*	*EI*	*TO*
	0.8752	0.7982	0.7739	0.7518
Guangxi	*RGDP*	*EI*	*UL*	*TO*
	0.6593	0.6508	0.6401	0.5832
Chongqing	*RGDP*	*EI*	*UL*	*TO*
	0.7110	0.6771	0.6529	0.6073
Sichuan	*RGDP*	*UL*	*EI*	*TO*
	0.6876	0.6677	0.6560	0.6503
Guizhou	*UL*	*RGDP*	*TO*	*EI*
	0.8727	0.8398	0.7921	0.7607
Yunnan	*RGDP*	*UL*	*EI*	*TO*
	0.7985	0.7518	0.6810	0.6728
Shaanxi	*UL*	*RGDP*	*EI*	*TO*
	0.8287	0.7924	0.7780	0.6305
Gansu	*UL*	*RGDP*	*EI*	*TO*
	0.8990	0.8768	0.8683	0.6695
Qinghai	*RGDP*	*EI*	*UL*	*TO*
	0.8364	0.7775	0.7649	0.5597
Ningxia	*UL*	*EI*	*RGDP*	*TO*
	0.8253	0.7891	0.7884	0.7177
Xinjiang	*RGDP*	*UL*	*EI*	*TO*
	0.8989	0.8694	0.8538	0.6977

**Table 2:** The hypothetical results of the panel environmental Kuznets curve (EKC) model.

$\beta_{1}$	$\beta_{2}$	$\beta_{3}$	Line-Type
$\beta_{1}>0$	$\beta_{2}<0$	$\beta_{3}>0$	N
$\beta_{1}<0$	$\beta_{2}>0$	$\beta_{3}<0$	Inverted-U
$\beta_{1}=0$	$\beta_{2}>0$	$\beta_{3}<0$	U
$\beta_{1}=0$	$\beta_{2}<0$	$\beta_{3}>0$	Inverted-U
$\beta_{1}=0$	$\beta_{2}=0$	$\beta_{3}>0$	Upward sloping straight line
$\beta_{1}=0$	$\beta_{2}=0$	$\beta_{3}<0$	Downward sloping straight line
$\beta_{1}=0$	$\beta_{2}=0$	$\beta_{3}=0$	None

**Table 3:** Panel EKC-estimated results in three regions.

Variables	Eastern China	Central China	Western China
Ln*RGDP*^3^	−0.156 ***	−0.140 ***	−0.107 ***
	(−5.69)	(−5.01)	(−3.81)
Ln*RGDP*^2^	4.495 ***	3.923 ***	2.895 ***
	(5.50)	(5.08)	(3.78)
Ln*RGDP*^1^	−42.39 ***	−36.08 ***	−25.26 ***
	(−5.22)	(−5.07)	(−3.64)
Ln*EI*	0.653 ***	0.271 ***	0.540 ***
	(10.45)	(5.32)	(3.21)
Ln*UL*	1.051 ***	0.546 ***	−0.226 *
	(9.72)	(6.08)	(−1.92)
Ln*TO*	−0.202 ***	0.101 ***	−0.144 ***
	(−4.97)	(3.47)	(−4.42)
$\alpha_{i}$	127.7 ***	107.9 ***	70.93 ***
	(4.78)	(4.94)	(3.36)
R^2^	0.8973	0.9491	0.877
Number of province	11	8	11
Observations	242	176	242
F	63.14	603.47	122.56

Note: The values in parentheses represent *t*-value; *, **, *** are expressed by *t*-test lower than 0.1, 0.05 and 0.01 significance levels, respectively.

**Table 4:** Inflection points and line-type of the three regions.

Item	Solution	Eastern China	Central China	Western China
Inflection point	Ln*RGDP*_1_	8.31	8.18	7.3922
	Ln*RGDP*_2_	10.90	10.50	10.6451
Inflection point (original value)	*RGDP*_1_	4064.31	3568.85	1623.27
	*RGDP*_2_	54,176.36	36,315.50	41,986.36
Line-type		Inverted-N	Inverted-N	Inverted-N
